# Baricitinib for Alopecia Universalis Following Drug-Induced Hypersensitivity Syndrome/Drug Reaction with Eosinophilia and Systemic Symptoms

**DOI:** 10.5152/eurasianjmed.2026.261426

**Published:** 2026-06-16

**Authors:** Yuji Kan, Hisashi Uhara

**Affiliations:** Department of Dermatology, Sapporo Medical University School of Medicine, Sapporo, Japan

To the Editor,

A 50-year-old woman with a history of postoperative glioma developed confluent erythematous patches over the entire body, accompanied by fever and bilateral axillary lymphadenopathy. Laboratory findings revealed elevated transaminase levels and eosinophilia. A drug-induced lymphocyte stimulation test demonstrated a 2.5-fold increase in lymphocyte proliferation in the presence of sodium valproate, which had been prescribed as postoperative epilepsy prophylaxis 3 months earlier. We confirmed that the patient was not taking any medications other than valproic acid that could have triggered drug-induced hypersensitivity syndrome/drug reaction with eosinophilia and systemic symptoms (DIHS/DRESS). Based on these findings, she was diagnosed with DIHS/DRESS. Although DRESS syndrome typically develops 2-6 weeks after the initiation of the causative drug, in this case it occurred 3 months after treatment initiation and was therefore considered delayed-onset DRESS syndrome.

The patient was treated with oral corticosteroids at a dose of 1 mg/kg/day, which were gradually tapered without recurrence of systemic symptoms. After 8 months, the dose was reduced to 10 mg/day, accompanied by a marked decrease in thymus and activation-regulated chemokine levels, from 94 540 pg/mL to 4225 pg/mL. Nine months after onset, the patient developed complete scalp hair loss, including the eyebrows and eyelashes, and complained of scalp pruritus. Alopecia universalis (AU) developed during the clinical course following DIHS/DRESS. These symptoms prevented further tapering of oral corticosteroids.

Various treatments, including cyclosporine at 3 mg/kg/day administered for 1 year, were initiated 1 week after the onset of DIHS/DRESS but proved ineffective. In contrast, treatment with baricitinib at 4 mg/day resulted in visible hair regrowth within 12 weeks of initiation. By week 48, approximately 90% of the scalp hair had regrown, and no significant adverse effects were observed. In addition, the patient’s concerns regarding steroid-related changes in appearance were resolved, as oral corticosteroids were successfully discontinued by week 48. Persistent scalp pruritus also markedly improved, with the numerical rating scale score decreasing from 10 at baseline to 1.

To our knowledge, this is the first report describing the marked efficacy of baricitinib in DIHS/DRESS complicated by AU. Alopecia associated with DIHS/DRESS should be considered in the differential diagnosis. In previously reported cases, alopecia occurred a mean of 46.5 days after the onset of DIHS/DRESS.^1^ Among these cases, 9 were treated with systemic corticosteroids and 1 with the Janus kinase 1 inhibitor abrocitinib.^2^

In the present case, hair loss persisted long after the onset of DIHS/DRESS and was refractory to corticosteroid therapy. Dermoscopic findings, including tapered hairs, black dots, and broken hairs, suggested that alopecia areata had newly developed during the disease course.

Recent studies have demonstrated that Janus kinase (JAK) 1/2 inhibitors, such as baricitinib, are effective in AU, likely through downregulation of cytokines including interferon-γ and interleukin (IL)-15.^3^ Drug-induced hypersensitivity syndrome/drug reaction with eosinophilia and systemic symptoms, in contrast, is a type 4 hypersensitivity reaction characterized by a T helper 2–predominant immune response involving eosinophil recruitment and activation mediated by cytokines such as IL-5, IL-6, IL-10, and IL-13, which play critical roles in disease pathogenesis.^4^^,^^5^

The shared involvement of cytokine signaling pathways suggests that JAK inhibitors may have therapeutic potential in both conditions. In the present case, baricitinib, a JAK1/2 inhibitor, may have exerted beneficial effects by modulating cytokine signaling relevant to both DIHS/DRESS and alopecia areata (AA) through the JAK–STAT pathway.
